# Heterogeneity of Microbial Communities in Soils From the Antarctic Peninsula Region

**DOI:** 10.3389/fmicb.2021.628792

**Published:** 2021-02-16

**Authors:** Pablo Almela, Ana Justel, Antonio Quesada

**Affiliations:** ^1^Department of Biology, Universidad Autónoma de Madrid, Madrid, Spain; ^2^Department of Mathematics, Universidad Autónoma de Madrid, Madrid, Spain

**Keywords:** microorgamisms, soil, distribution, heterogeneity, homogeneity, Antarctica

## Abstract

Ice-free areas represent less than 1% of the Antarctic surface. However, climate change models predict a significant increase in temperatures in the coming decades, triggering a relevant reduction of the ice-covered surface. Microorganisms, adapted to the extreme and fluctuating conditions, are the dominant biota. In this article we analyze the diversity and composition of soil bacterial communities in 52 soil samples on three scales: (i) fine scale, where we compare the differences in the microbial community between top-stratum soils (0–2 cm) and deeper-stratum soils (5–10 cm) at the same sampling point; (ii) medium scale, in which we compare the composition of the microbial community of top-stratum soils from different sampling points within the same sampling location; and (iii) coarse scale, where we compare communities between comparable ecosystems located hundreds of kilometers apart along the Antarctic Peninsula. The results suggest that in ice-free soils exposed for longer periods of time (millennia) microbial communities are significantly different along the soil profiles. However, in recently (decades) deglaciated soils the communities are not different along the soil profile. Furthermore, the microbial communities found in soils at the different sampling locations show a high degree of heterogeneity, with a relevant proportion of unique amplicon sequence variants (ASV) that appeared mainly in low abundance, and only at a single sampling location. The Core90 community, defined as the ASVs shared by 90% of the soils from the 4 sampling locations, was composed of 26 ASVs, representing a small percentage of the total sequences. Nevertheless, the taxonomic composition of the Core80 (ASVs shared by 80% of sampling points per location) of the different sampling locations, was very similar, as they were mostly defined by 20 common taxa, representing up to 75.7% of the sequences of the Core80 communities, suggesting a greater homogeneity of soil bacterial taxa among distant locations.

## Introduction

Ice-free areas in Antarctica comprise less than 1% of the continent (Cowan, 2014; Burton-Johnson et al., 2016), constituting extremely cold and arid distant and isolated patches within a matrix of ice. These areas, which are not permanently covered by snow or ice, considered oases in the middle of a desert, are of an enormous ecological relevance, since are home to most of the continent’s biodiversity.

Antarctica is warming. Although the rate of warming in maritime Antarctica seems to be slowing down (Turner et al., 2016), Rignot et al. (2019) has determined an ice mass loss of billions of tons per year, for the period 1979 to 2017, in all regions of the continent due to climate change. The Antarctic Peninsula region has had the largest warming of any other terrestrial environment in the southern hemisphere in recent decades (Siegert et al., 2019). The predictions for the end of the century suggest a 25% increase of new ice-free areas in Antarctica, with more than 85% emerging in the North Antarctic Peninsula bioregion (Lee et al., 2017).

These ice-free areas have been exposed for a variable time span, being subjected to glacier retreats and advances. Thus, some areas have been only recently deglaciated and exposed for some decades, as Clark Nunatak (Oliva and Ruiz-Fernández, 2017), while others have been mostly deglaciated for millennia, as Byers Peninsula (Livingston Island, Antarctic Peninsula region) (Oliva et al., 2016). They include islands, nunataks (exposed mountain tops), cliffs, plateaus, ice-free valleys and scree slopes, among others. In any of its forms, these ecosystems are governed by low temperatures, wide temperature fluctuations, low nutrient status, low water availability, high incident radiation, and high levels of physical disturbance (e.g., glaciofluvial activity, frost weathering and cryoturbation). These extreme conditions preclude the establishment of larger organisms (macrobes), resulting in environments dominated by microorganisms (Hughes et al., 2015). Therefore, they constitute a perfect scenario for the study of distribution establishment and ecological functioning of soil microbial communities.

Although new ‘omics’ techniques have contributed greatly to a better understanding of communities inhabiting soils (Smith et al., 2006; Krauze et al., 2020), this knowledge is quite fragmented, and results obtained from different studies are hardly comparable among them to obtain a clear idea about the distribution of microbial communities in polar regions. It is well known in other latitudes that the microbial communities are different along the soil profiles, with relevant heterogeneity in the distribution of the soil microorganisms in the same biotope (Frey et al., 2013). The highest abundance and diversity of microorganisms that inhabit soils are located in the most superficial centimeters (Brown and Jumpponen, 2014). Previous studies have examined the effects of depth on Antarctic soil bacterial communities (Herbold et al., 2014). Nevertheless, this heterogeneous distribution of the microbial communities has not been as widely studied on a wide geographical scale (Horrocks et al., 2020), where cryoturbation and other physical process can alter the biotope.

In this paper we analyze the composition of the soil bacteria at three different scales, in order to determine at what level of sampling we were able to identify heterogeneity between ecologically comparable soils. At the fine scale, we compare the microbial community differences between the top-stratum soil (tss) and deeper-stratum soils (dss) at exactly the same sampling point at two sampling locations. In the medium scale, we study the heterogeneity of bacterial communities in top stratum soils at a local scale, comparing the diversity and structure of the communities obtained in sectors within the same geographical location. Finally, in the coarse scale we compare communities from the top stratum soils among sampling locations located in a wide range in the Antarctic Peninsula region.

Our main working hypothesis is that in extreme ecosystems, such as Antarctic soils, in which environmental constraints are the limiting factors, some microorganisms with better survival strategies and better dispersal mechanisms, will occur in those communities without reference to geographical distribution, or local minor aspects. Therefore, microbial communities will present common taxa in ecologically comparable ecosystems even at wide geographical scale.

## Materials and Methods

### Study Area

**FIGURE 1:**
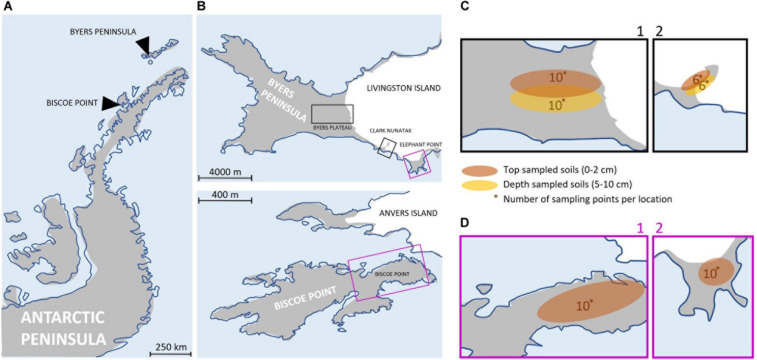
**(A)** Map of the Antarctic Peninsula. **(B)** Maps showing the 4 sampling locations at Byers Peninsula and Elephant Point in Livingston island (South Shetland Islands) and Biscoe Point (Anvers Island). The sampling locations have been highlighted by squares of different colors, depending on whether the samplings were of top-stratum soils (0–2 cm; purple square) or top- and depper-stratum soils (0–2 cm/5–10 cm; black square). **(C)** Local-scale sampling locations where top- and depper-stratum soils samples were collected: Plateau (C1) and Nunatak (C2). The numbers indicate the samples that were collected. **(D)** Local-scale sampling locations where top-stratum soil samples were collected: Biscoe (D1) and Elephant (D2). The numbers indicate the samples that were collected.

The study area included four locations along the Antarctic Peninsula (Figure 1A). These areas were selected based on their ecological similarity, remaining ice-free during the summer and vegetation free and free of megafauna disturbances, to the best of our knowledge (Supplementary Plate 1). The physical and chemical characteristics of the soil were similar among locations (Supplementary Table 1), with values of Total Organic Matter (TOM) and C/N ranging between 0.2–0.3%(p/p) and 3.8–5, respectively. The apparent density of soils showed equal values for all locations (1.6 g/cm^3^), and the soil texture classification was determined as ‘sandy loam’ and ‘loamy sand.’ The pH values showed variations that ranged between 4.6 and 7.9.

There are evidences indicating that the four sampling locations were ice covered for different time periods (from millennia to decades), following next gradient from the oldest to the most recent: Byers Plateau, Elephant Point, Clark Nunatak, and Biscoe Point.

Three sampling locations were located in Livingston Island, the second largest island in the South Shetland archipelago: Byers Plateau, Clark Nunatak and Elephant point. Byers Peninsula, located at the western end of Livingston Island, is one of the largest ice-free areas in the Antarctic Peninsula and the largest in the South Shetland Islands. The coast itself and the ice margin of Rotch Dome glacier form a clearly defined and visually obvious boundary with the island, of approximately 60 km^2^. The retreat of Rotch Dome glacier pre-dated 8.3 cal. ky in the westernmost third of the peninsula as a response to warmer climate conditions in the Antarctic Peninsula region during the Early Holocene, and continued eastwards, becoming ice-free the easternmost area probably before 1.8 cal. Ky (Oliva et al., 2016). Today the Rotch Dome sits in contact with the moraine in the central plateau, therefore not being associated to a recent process of glacial retreat. Soils from Byers Plateau (62°38′S, 60°58′W, Supplementary Table 2) were collected from an area of the plateau bordering the Rotch dome glacier front and moving away up to 500 m (Figure 1B).

Clark Nunatak is a rocky peak located in the SE corner of Byers Peninsula, surrounded by the Rotch Dome glacier. It is estimated that the glacier has retreated from the moraine limits very recently, probably after 1950 (Oliva and Ruiz-Fernández, 2017; Palacios et al., 2020). For this reason, it is not until 2002 when the Antarctic Treaty Consultative Meeting (ATCM) included it within ASPA 126, since in previous versions the small ice-free ground surface did not exist. Samples from Nunatak (62°40′S, 60°54′W, Supplementary Table 2) were collected from an area bordering the Rotch Dome glacier front and moving away up to 200 m (Figure 1B). Elephant Point (E) is an ice-free peninsula of 1.16 km^2^ in the SW of Livingston Island. It is limited by the Rotch Dome glacier in the north and the sea encircling the rest of its margins. In this area there is evidence of glacial retreat, which has been accelerated over the last decades in response to the recent warming detected in the Antarctic Peninsula region (Steig et al., 2009; Thomas et al., 2009). It is estimated that 17% of the total land surface exposed today in Elephant Point appeared between 1956 and 2010 (Oliva and Ruiz-Fernández, 2015). Soil samples from Elephant Point (62°40′S, 60°51′W, Supplementary Table 2) were collected from an area bordering the Rotch Dome glacier front and moving away up to 300 m (Figure 1B).

The fourth sampling location was at Biscoe Point, an area of 0.59 km^2^ located near the south-west coast of Anvers Island, in the Palmer Archipelago. Until recently, Biscoe Point formed a peninsula joined to Anvers Island by an ice ramp extending from the adjacent glacier. The ice ramp disappeared as the glacier retreated at least between 1985 and 2004 (ATCM XIII,1985; ATCM, 2004), and a narrow sea channel now separates Anvers Island from the island on which Biscoe Point lies (ATCM, 2014). Soil samples from Biscoe (64°48′S, 63°46′W, Supplementary Table 2) were collected from the area closest to the glacier front, now on Anvers Island, and moving away up to 400 m (Figure 1B).

### Sampling

Samplings were conducted during two different Antarctic campaigns: February 2018 in Plateau and Nunatak (Figure 1C) and January 2019 in Biscoe and Elephant (Figure 1D). The sampling points were fixed previously to the field campaign to collect in each location two samples from each of the 5 sectors (from I to V) in Plateau, Elephant and Biscoe, and 3 sectors (from I to III) in Nunatak (Supplementary Table 2), in order to test the potential heterogeneity due to patchy distribution of bacterial soil communities.

At each sampling point, we obtained samples comprised of 3 subsamples collected within at approx. 1 m distance of top-stratum soils (0–2 cm) to avoid the vertical heterogeneity in microbial communities attributable to soil horizon development, as recommended by Sigler et al. (2002) and Rime et al. (2015). It is in these first few centimeters that factors such as light, among others, could be critical for the C input in the ecosystem, and conditioning the composition of the communities. The soils considered into this study do not have another organic C input than microbial primary production (i.e., photosynthesis), since there are no plants and animal debris cannot reach the sites, besides the aerial transportation and eventual birds droppings. Additionally, in order to test the fine scale homogeneity due to the soil horizons development, deeper-stratum soil (5–10 cm) were collected in each sampling point of Plateau and Nunatak.

All soil samples were placed in sterile 50 ml Falcon^®^ tubes and frozen at −20°C for shipment and storage until processing in the laboratory. Every sample was obtained directly with the plastic tubes without any tool to avoid potential contamination.

### DNA Extraction, Sequencing and Taxonomical Assignment

Total genomic DNA extraction was performed independently from the 4 different sampling locations and for soil strata, using the PowerSoil DNA Isolation Kit (MO BIO Laboratories, Inc.) according to standard procedures. DNA concentrations were determined in a NanoDrop ND 1000 spectrophotometer (Thermo Fisher Scientific^TM^). The 16S rRNA gene was amplified by PCR using barcoded primers set 341F (5′- CCT AYGGGRBGCASCAG -3′) and 806R (5′- GGACTACNNGGG TATCTAAT -3′) targeting the V3–V4 hypervariable regions (Otani et al., 2014). This universal primer set is for bacterial community and the archaeal community was not included in the study. The pool of samples with the prepared libraries was sequenced by Illumina MiSeq platform. The sequencing was performed in two cycles, in the first cycle the Plateau and Nunatak samples were included, and in the second cycle the Elephant and Biscoe samples.

Microbiome 16S rRNA gene diversity was assessed with QIIME v2-2019.4 (Bolyen et al., 2019). Briefly, cleaned and trimmed paired reads were filtered and denoised using DADA2 plug-in (Callahan et al., 2016). For chimera identification, 250.000 training sequences were used. Identified amplicon sequence variants (ASVs) were aligned using MAFFT (Katoh et al., 2002) and further processed to construct a phylogeny with fasttree2 (Price et al., 2010). Taxonomy was assigned to ASVs using the q2-feature-classifier (Bokulich et al., 2018) and blasted against the SILVA v132 99% 16S sequence database (Quast et al., 2012). Taxonomical assignation was carried out at the same time with all samples after the bioinformatics took place.

Sequences generated by this study were deposited to GenBank under the BioProject accession number PRJNA678471.

### Statistical Analysis

Some summary statistics of the ASVs obtained in each sampling location were calculated to obtain ‘Total ASV’ (total quantity), ‘Different ASV,’ ‘Predominant ASV’ (more than 1000 copies per sampling location) and ‘Unique ASV’ (present in a sampling location and absent in the others). Also, ASVs were binned into ‘core community’ (highly persistent) if present in 90% (referred to as the Core90) or 80% (Core80) of soil samples. The Core90 community analysis was carried out for Plateau, Nunatak, Elephant and Biscoe sampling locations together. The Core80 community analysis was performed for the 4 sampling locations separately, thus obtaining the ‘core community’ of each location (if present in 80% of sampling points of a sampling location). The data obtained from each Core80, and their taxonomic assignments, were jointly compared between the 4 locations to determine their distributions. Results are shown in Table 2.

Alpha diversity indices (Richness and Shannon Index) and their rarefaction curves were estimated using the plugin q2-diversity (running 10 iterations and 1000 sequence steps up to the maximum number of sequences per sample). The lowest sample-specific sequencing depth (104777) were used to compensate for the variation in read numbers. Beta diversity was assessed using Bray-Curtis dissimilarities between the community compositions of the sampling sites.

We have proposed different experimental design models, depending on the scale, for analyzing the influence of the depth, sector or location factors on the diversity:

- At the fine scale, we adjusted two balanced block experimental design models in which the factor was the soil depth, and the block was the sampling point. One with data from 2 soil depths at 10 sampling points in Plateau, and the other with data from 2 soil depths at 6 sampling points in Nunatak.
- At the medium scale, we adjusted four balanced one-factor experimental design models, one for each location (Plateau, Nunatak, Elephant, and Biscoe), in which the sector was the factor. Each model was fitted with data from two top-stratum soils of each of the sectors into which the sampling locations were divided.
- At the coarse scale, we adjusted a one-factor experimental design model, in which the factor was the location, with the data from all the top-stratum soils collected at the four locations.

We tested the homogeneity of alpha diversity indices between the two soil horizons, within site locations, and between locations, using one- and two-way ANOVA tests. To make the same comparisons using the information provided by the Bray-Curtis dissimilarity matrices, we used permutational multivariate analysis of variance tests (PERMANOVA). Differences are considered statistically significant if *p*-value < 0.05. When any of the hypothesis of equality of means is rejected, the corresponding multiple comparisons are made with Bonferroni correction with overall significance level α = 0.05.

Plots of the two principal components of PCoA were used to visualized proximity in the community composition among samples. A heatmap of two-way cluster using Bray-Curtis dissimilarities was used to visualize the relative abundances of bacteria in Core80 of Plateau, Nunatak, Elephant and Biscoe. The heatmap was based on the predominant ASVs (≥1000 copies) present in any of the Core80.

Data visualization, alpha and beta diversity comparisons and multivariate statistics were performed using the R environment with the vegan (Oksanen et al., 2015) and ggplot packages.

## Results

### Diversity and Microbial Community at Different Soil Strata (Fine Scale)

**FIGURE 2:**
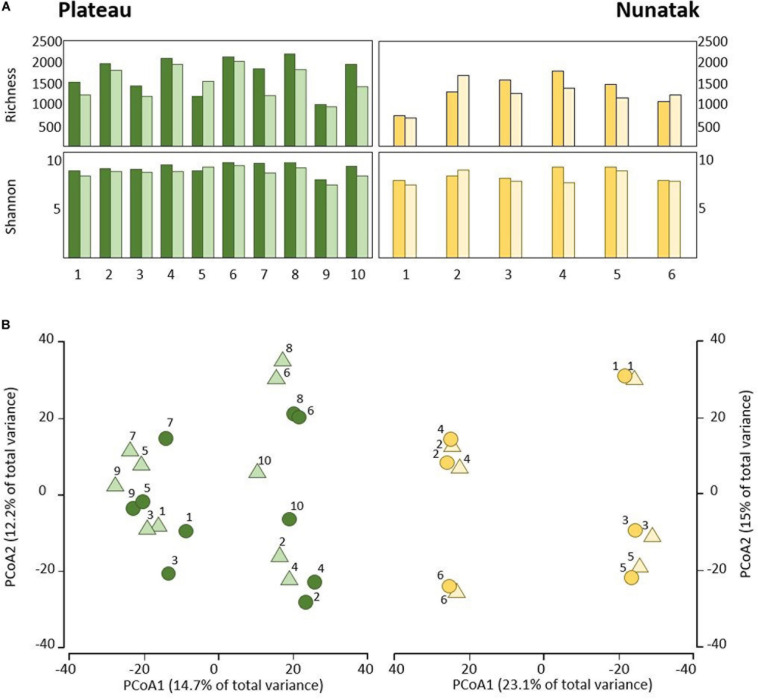
**(A)** Alpha diversity indices of the bacterial community of top-stratum soils (0–2 cm; dark colored plots) and deeper-stratum soils (5–10 cm; light colored plots) from Plateau and Nunatak. **(B)** Principal coordinate analysis (PCoA) of the bacterial community composition of top-stratum soils (0–2 cm; dark colored dots) and deeper-stratum soils (5–10 cm; light colored triangles) from Plateau and Nunatak. The principal coordinate analysis are based on Bray-Curtis dissimilarity matrices between the microbiome profiles. Distances between symbols on the ordination plot reflect relative dissimilarities in community structures. The variation in microbial community structures explained by each PCoA axis is given in parentheses.

Our sampling design focuses first on identifying the differences between the bacterial community in the two soil-strata defined at two sampling locations (Plateau and Nunatak). The alpha diversity indices have shown a markedly different pattern between the sampling locations (Figure 2A). At the sampling location Nunatak, there were no significant differences between the means of the Richness (1328 and 1238 for tss and dss, respectively) and Shannon indices (8.5 and 8.1 for tss and dss, respectively) in the communities found at the two soil-strata (Table 1). On the contrary, the means of both alpha diversity indices were significantly different at Plateau sampling location. The mean Richness in tss was 1729 (SD = 422), higher than in dss, where it was 1512 (SD = 373). The mean of Shannon index in tss was 9.4 (SD = 0.6), also higher than in dss, where it was 8.9 (SD = 0.6). Similar behavior was observed in the bacterial community composition. The PERMANOVA tests (Table 1) showed that there were significant differences between tss and dss communities in Plateau (*p* = 0.007), while the differences were not significant in the soil profile at Nunatak (*p* = 0.814).

**TABLE 1:** *p*-values of the ANOVA and PERMANOVA (Permutational Multivariate Analysis of Variance) tests for comparisons of richness and diversity, and composition of the bacterial community, respectively, among surface soil samples at different areas in each location (Plateau, Nunatak, Elephant, and Biscoe).

Scale	Description of the samples	Richness	Diversity	Community	ANOVA/Permanova	Sample size
Fine	Top-stratum soils (0–2 cm) and deeper-stratum soils (5–10 cm) from Plateau	**0.034**	**0.005**	**0.007**	Two-way	10 pairs
Fine	Top-stratum soils (0–2 cm) and deeper-stratum soils (5–10 cm) from Nunatak	0.517	0.259	0.815	Two-way	6 pairs
Medium	top-stratum soils (0–2 cm) from Plateau	0.753	0.452	0.657	One-way	2 samples for 5 areas
Medium	top-stratum soils (0–2 cm) from Nunatak	0.205	0.832	0.800	One-way	2 samples for 3 areas
Medium	top-stratum soils (0–2 cm) from Elephant	0.990	0.590	**0.012**	One-way	2 samples for 5 areas
Medium	top-stratum soils (0–2 cm) from Biscoe	0.208	0.149	0.380	One-way	2 samples for 5 areas
Coarse	Top-stratum soils (0–2 cm) from Plateau (P), Nunatak (N), Elephant (E), and Biscoe (B)	0.099	**0.003**	**0.000**	One-way	10 samples of P, 6 of N, 10 of E, 10 of B

**TABLE 2:** Taxonomy summary of the dominant bacteria in Core80 analysis of the sampling locations.

Taxa	Plateau	Nunatak	Elephant	Biscoe
	% Core80	% Core80	% Core80	% Core80
Hydrogenophilaceae (Thiobacillus)	–	–	1.7	0.4
Iamiaceae (Iamia)	0.7	0.4	1.4	0.7
Tenderiaceae (Tenderia)	–	1.2	7.1	0.2
Pseudanabaenaceae (Pseudanabaena)	–	4.1	0.8	0.3
Phormidiaceae (Tychonema)	–	0.0	6.7	10.9
Intrasporangiacea (Oryzihumus)	11.8	9.9	3.6	1.2
Gaiellales	14.4	2.4	2.5	1.6
Chitinophagaceae (Chitinophaga)	7.1	5.3	5.0	7.4
Gemmatimonadetes (Gemmatimonas)	12.0	21.9	3.2	4.9
Chthoniobacteraceae (Udaeobacter)	7.0	0.5	–	–
Actinobacteria (MB-A2-108)	2.8	0.7	–	–
ilumatobacter (Ilumatobacter)	3.1	2.4	4.2	4.5
Frankiales	2.5	0.6	2.3	2.4
Chloroflexi- KD4	2.2	1.9	1.8	1.2
Pyrinomonadaceae (RB-41)	1.8	0.1	–	–
Sphingomonadaceae (Sphingomonas)	4.4	12.3	13.2	17.9
IMCC26256 (Ferrimicrobium)	2.1	0.8	0.1	–
Nocardioidaceae (Nocardioides)	1.5	1.1	4.7	2.6
Xanthomonadaceae (Lysobacter)	1.4	7.2	5.0	7.4
Rubrobacteria	1.4	0.1	1.4	2.3
Solibacteraceae (Bryobacter)	1.3	0.2	0.6	0.4
Blastocatellaceae (JGI 0001001-H03)	1.8	0.6	0.7	3.0
Burkholderiaceae (Burkholderia-Caballeronia-Paraburkholderia)	1.6	3.2	4.3	5.2
Holophagae	1.0	0.3	0.2	0.4
Rhodobacteraceae	0.0	0.2	0.7	1.4
Rhizobiales + Xanthobacteraceae	1.4	1.0	1.3	1.3
Hymenobacteraceae (Hymenobacter)	0.0	0.0	1.5	1.0
Rhodanobacteraceae (Rhodanobacter)	0.4	0.9	0.7	1.0
Nitrosomonadaceae (Nitrosomonas)	1.0	1.5	1.1	0.0
Acetobacteraceae	0.7	0.4	0.2	0.0
Micrococcaceae	0.7	3.4	1.0	1.5
Total%	86.0	84.8	77.1	81.0

*The total number of sequences belonging to the Ce80 communities ranged from 120209 in Nunatak to 500390 in Biscoe.*

The differences between tss and dss community structures were smaller at Nunatak than at Plateau. The PCoA plot (Figure 2B) illustrates these two different results, showing closer similarity between samples in the same sampling point in the case of Nunatak. In Plateau, samples from the same sampling point are as similar as those from different sampling points at the same or different depths points.

### Diversity and Composition of Prokaryotic Communities Within the Different Sampling Locations (Medium Scale)

The bacterial communities from top-stratum soils from Plateau, Nunatak, Elephant and Biscoe locations were analyzed independently for each sampling location. There were not significant differences in terms of richness, diversity and community structure (Table 1). ANOVA and PERMANOVA tests showed no significant evidence of within location heterogeneity when comparing the different sampling sectors, with the glacial front of each site as a reference point, considered in Plateau, Nunatak and Biscoe. In location Elephant there were no significant differences between the sampled sectors in terms of richness and diversity; although a significant evidence of heterogeneity (PERMANOVA test *p* = 0.012) was observed in the structure of the prokaryotic communities among the sampled sectors.

### Bacterial Community Diversity, Richness and Structure at Different Locations (Coarse Scale)

**FIGURE 3:**
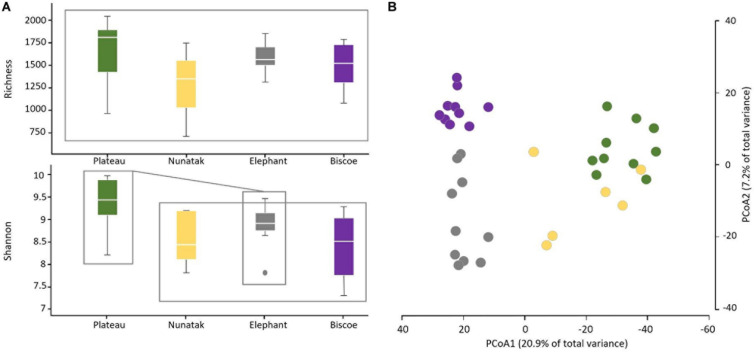
**(A)** Boxplots of the alpha diversity indices of the bacterial community composition of top-stratum soils (0–2 cm) for each of the sampling locations included in this study. **(B)** Principal coordinate analysis (PCoA) of the bacterial community composition of top-stratum soils (0–2 cm) from the samplings. The principal coordinate analysis is based on Bray-Curtis dissimilarity matrix. Distances between symbols on the ordination plot reflect relative dissimilarities in community structures. The variation in microbial community structures explained by each PCoA axis is given in parentheses.

From the results obtained in the medium scale, we can consider all the sampling sites within the same location as a representative sample of its soil microbial community. Comparing the four locations, we observe that median of ASVs richness and diversity was the highest in the Plateau samples and lowest in the Nunatak ones (Figure 3A). ANOVA and PERMANOVA tests (Table 1) indicated that both the diversity and composition of the bacterial community were different between locations, while there were no significantly clear differences between the means of Richness. For the Shannon index, the Bonferroni multiple comparison tests showed that there were only significant differences between the Plateau mean and the Elephant and Biscoe means. There was no significant evidence of heterogeneity in the comparisons among Nunatak, Elephant and Biscoe, neither between Plateau and Elephant (Figure 3A).

The differences in the bacterial communities measured with the Bray-Curtis dissimilarity are represented in the plot of the two main principal coordinates obtained in a PCoA (Figure 3B). Ordinations based on this metric demonstrated a clear separation of bacterial communities among the sampling locations, except for the bacterial community from Nunatak which was slightly intermixed with Plateau and midway toward the other two communities, Biscoe and Elephant.

### Description of Microbial Communities and Similarities in Communities Among Sampling Locations

**FIGURE 4:**
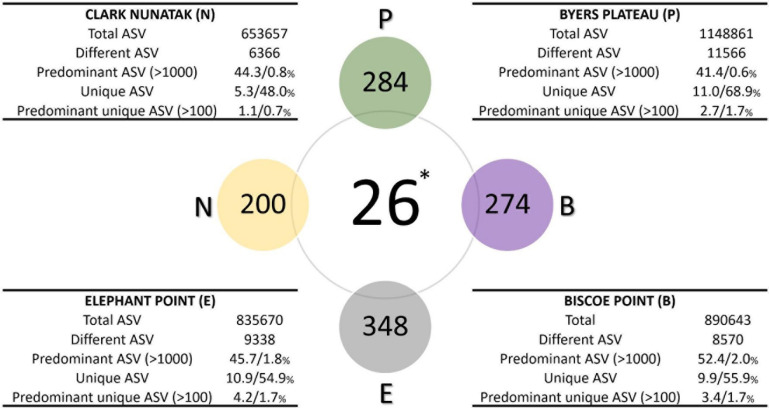
Diagram comparing the bacterial core communities. Colored spheres indicate the number of ASVs in at least 80% of top-stratum soils (core80) of each sampling location, while the central sphere (^∗^) indicates the Core90 (90% of all the top-stratum soils). For each location, in the tables are shown the number of total and different ASV of the bacterial community, and the percentages of the predominant ASV (at least 1000 copies), the unique ASV at each location (not present in other sampling areas), and the percentage of the predominant unique ASV (at least 100 copies), according to total and different ASV, respectively.

The description of the communities found in the different sampling locations is illustrated in Figure 4. In Plateau the highest number of different ASV was found, which was almost 2-fold higher than the community from Nunatak location (11566 and 6366 ASVs for Plateau and Nunatak, respectively), with the lowest amount of different ASVs. At Plateau location only 0.6% of the different ASVs (70 ASVs) were found predominant with more than 1000 copies and represented over 41% of the total ASVs, indicating that a low number of different sequences represented a large proportion of the community found at this location. Moreover, at Plateau location 11% of the total sequences were found only at this sampling location (unique ASVs) and reached 68.9% of the different sequences. Most of those unique sequences are found in low abundance, thus, only 2.7% of the total sequences were unique and predominant (over 100 copies) representing 1.7% of the different ASVs. A very similar pattern was found in the other locations, where the number of predominant ASVs (more than 1000 copies) reached 44, 45.7 and 52% of the total ASVs for Nunatak, Elephant and Biscoe, respectively. These predominant sequences were composed of few different ASVs, representing 0.8, 1.8, and 2% of the total different ASVs for Nunatak, Elephant, and Biscoe. In addition, 5.3, 10.9 and 9.9% of the total ASVs found in Nunatak, Elephant and Biscoe were classified as unique ASVs, representing 48, 54.9 and 55.9% of the diversity of the different ASVs determined in each location.

### Core Community

The microbial Core90 analysis revealed that 26 ASVs were found in at least 90% of the total sampling points from all locations (Figure 4). These results indicated that the soil bacterial communities at the highest level of taxonomic resolution were apparently not homogeneous between the 4 sampling locations. This ‘core community’ represented a low proportion of ASVs from total ASVs sequenced.

**FIGURE 5:**
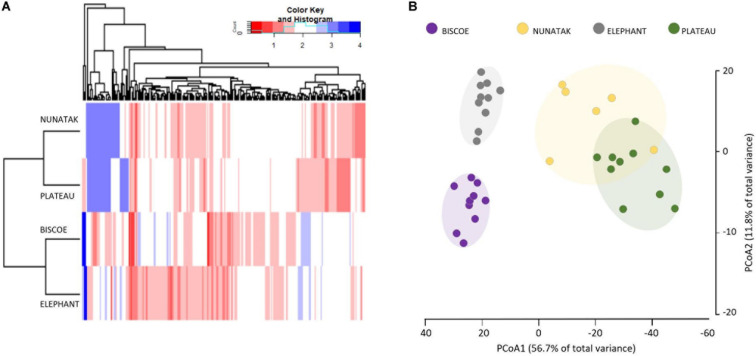
**(A)** Heatmap of two-way cluster analysis performed on most abundant Core80 bacterial community (only ASVs > 1000 copies) of all top-stratum sample soils from Plateau, Nunatak, Elephant and Biscoe, using Bray-Curtis dissimilarities. The color intensity in the cluster dendrogram correspond to the abundance of normalized reads. **(B)** Principal coordinate analysis (PCoA) for all the sampling points when considering only the ASVs in any of the Core80. The principal coordinate analysis is based on the Bray-Curtis dissimilarity matrix. Distances between symbols on the ordination plot reflect relative dissimilarities in community structures. The variation in microbial community structures explained by each PCoA axis is given in parentheses.

The ‘Core80’ was analyzed for Plateau, Nunatak, Elephant, and Biscoe, to describe its local composition and relative abundances (Figure 4). Thus, 284 ASVs in Plateau, 200 ASVs in Nunatak, and 348 and 274 ASVs in Elephant and Biscoe, respectively, were revealed. A PCoA plot, based on Bray-Curtis dissimilarity, was generated (Figure 5B) to visualize the proximities between the sampling points when considering only the ASVs found in any of the Core80. Results showed that ASVs from the four Core80 bacterial communities are well separated, except for one sample from Nunatak that is closer to some samples from Plateau than to the rest. A Heatmap (Figure 5A) was used to visualize the relative abundance of predominant ASVs (≥1000 copies) from Core80, regardless of taxonomic assignment. Results revealed that Plateau and Nunatak clustered together and separately from Elephant and Biscoe, that also clustered together, suggesting that core communities from those locations have more in common than the other locations.

The Core80 communities were also explored in detail in terms of taxonomic assignment. Family level was considered appropriate because of taxonomic resolution and ecological relevance for most of the bacterial groups. At this taxonomic level (or similar) the core communities were represented by a much lower number of identities, which ranged from 25 to 28. The ASVs included in those groups represented between 77.1 and 85.4% of the total sequences within the Core80 community. The most part (20) of the identities were found in all the core communities and represented up to 75.7% of the total ASVs in the Core80. These 20 groups belonged in the majority to Actinobacteria (8) and Proteobacteria (6), but Acidobacteria, Gemmatobacteria, Bacteriodetes, Cyanobacteria, and Chloroflexi were also represented (Table 2).

## Discussion

Microorganisms are the dominant biota and play a key role in the ecology of Antarctic terrestrial ecosystems (Cowan, 2014). Investigating the distribution of microbial diversity is essential to understanding ecological functioning of these ecosystems. The purpose of our analysis is to investigate the heterogeneity of the microbial community inhabiting ecologically comparable soils (no vegetation in the proximity of the sampling site, no megafauna presence, similar microtopographic characteristics, granulometry, etc.) in the Antarctic Peninsula. This work considers different scales, starting at the fine scale, in which we compare, at the closest sampling locations (Plateau and Nunatak), the microbial community differences between the top-stratum (tss) and deeper-stratum soils (dss) at exactly the same sampling point. The medium scale brings into comparison the top-stratum communities found within the same sampling location (e.g., Plateau). Finally, the coarse scale compares the top stratum communities found among the four sampling locations (Plateau, Biscoe, Nunatak and Elephant) with diverse geographical distance.

The study revealed that comparisons of bacterial communities from different soil strata at Plateau resulted in low P-values (*P* = 0.007), indicating significant differences among the soil-strata, and therefore a non-homogeneous and differentiated distribution according to a vertical profile. Conversely, bacterial populations from Nunatak have shown a vertical homogeneity of their communities. These differences in the homogeneity of distribution among soil strata in the two locations are attributable to the length of the ice-free period, since ecologically the sampling locations are comparable. Oliva et al. (2016) have dated areas close to the soils studied in Byers Peninsula in thousands of years, while the soil samples from Nunatak Clark have only been ice-free in summer for a few decades from the present. As described by Barrett et al. (2006), vertical distributions of the soil microbiomes would be related to the variation in soil properties. Niederberger et al. (2008) showed how the highest microbial diversity in Antarctic soils was related with the highest levels of organic matter, while Chu et al. (2016) also related it to the N available in the different soil layers of the Tibetan Plateau. Such large temporal differences among Plateau and Nunatak should differentiate both locations in terms of soil properties established along the vertical profile. Therefore, the relative homogeneity in Nunatak among their prokaryotic communities would be related to the uniform characteristics of the soils, both in surface and depth. The opposite is shown in Plateau, where the microbial activity, over time, would have contributed by modifying the characteristics of the soil. Shannon diversity analysis indicated significant differences in microbial diversity between soil strata in Plateau (9.4 tss and 8.9 dss) and did not indicate relevant differences in Nunatak (8.5 tss and 8.1 dss). For both locations, bacterial communities in top-stratum soils have shown greater richness and diversity values than those from the deeper-stratum soils. Therefore, our results are similar to those reported from other Antarctic soils (Aislabie et al., 2006; Bajerski and Wagner, 2013), showing that the highest abundance and diversity of soil microorganisms are located in the most superficial centimeters.

Previous studies have shown high levels of spatial heterogeneity in prokaryote biodiversity across terrestrial environments in Antarctica (Barrett et al., 2006; Chong et al., 2010). This spatial heterogeneity is generated by physicochemical and trophic variations acting at all spatial scales. However, our results with the top-stratum soils of each sampling location showed no significant evidence of within locations heterogeneity, both for alpha diversity and community composition. The diversity of prokaryotes is sensitive to local environmental conditions such as the availability of water and nutrients (Barrett et al., 2006; Chong et al., 2010) and soil heterogeneity is expected in small spatial scales (Fraschetti et al., 2005). However, our data, from samples taken from carefully chosen ecologically comparable sites and locations, offer another view about the microbial compositions of soils at different scales of analysis. When all sequences from the sampling sites are analyzed jointly, data points from the same locations are closer supporting the heterogeneous distribution of the microbial communities. Differences in bacterial communities measured with Bray-Curtis provide insight into differences in community composition among samples, with the advantage of being based on ASV counts, regardless of taxonomic assignment as maximum sequencing resolution. The results showed that the bacterial communities from soils are again grouped by locations, suggesting that inter-site variations are greater than intra-site variations. Therefore, geological variables (i.e., spatial distance, climate conditions, geological features and historical context) are also of influence on microbial communities. These results are similar with previous studies such as that of Yergeau et al. (2007), which have reported significant differences in bacterial diversity in Antarctica on a continental scale.

Description of microbial communities from ASVs allows a different perspective of its composition, being able to analyze sequences with the maximum potential resolution, and with no risk of introducing deviations due to the taxonomic reference (Callahan et al., 2016). However, one of the costs of this method leads to a possible loss of real sequences that would be present at very low levels. Our data indicate that a large proportion of the different sequences are unique. The amount of these unique ASVs assigned to each location is high, ranging from 5.3 to 11% of the total sequences, having a key role in the heterogeneity of the communities. However, clustering thresholds greater than 97% identity can lead to an overestimation of the rare biosphere present in the samples (Kunin et al., 2010). Most of these unique ASVs are in low abundance but represent between 48% and 69% of the different sequences. At the moment, the ecological role of that rare biosphere is not well understood, but frequently neglected.

Analyses based on the core communities allow us to know the specific weight of those groups shared between different samples. The core at maximum resolution for the 4 sampling locations (Core90) makes up a common bacterial community in 90% of sampled soils, of 26 ASVs which do not dominate the set sequences. These data, similar to those obtained in previous studies of core communities from different Antarctic environments (Murray et al., 2020), show that at least a few dozen sequences are identical between ice-free areas separated by hundreds of kilometers along the Antarctic Peninsula. The possibilities of dispersal of microorganisms are numerous, ranging from air transport (Archer et al., 2019; King-Miaow et al., 2019; Cao et al., 2020) and ocean currents (Fraser et al., 2018), to the anthropogenic activities (Kirtsideli et al., 2018). The dispersal capabilities of bacteria are evidenced in Antarctica showing that identical sequences are found in relatively distant regions. However, the ASVs heterogeneity between locations in the Core80 community is significant. Although the sampling locations Plateau, Nunatak and Elephant are located at the same island with a maximum distance of 10 km, bacterial community from location Elephant, located at more than 300 km away from location Biscoe, is more similar to that one than to the closer ones.

The previous analyses used ASVs as an expression of diversity, without considering the taxonomic assignment of those sequences. However, when the taxonomy is the purpose, it is necessary to find a balance between the genotypic diversity and the functional diversity, making in this case relevant to reduce the taxonomic resolution for the analyses. The detailed taxonomic assignment of the Core80 communities indicated that a small number of taxa, at Family level, conformed the bacterial core communities (less than 30 taxa) of each location. On average, these groups represented over 82% of the sequences forming those core communities. In fact, 20 of those identities were present at all sampling locations and can be considered cosmopolitan taxa in this region and represented up to 75.7% of the total sequences found at the Core80 community. Soils from extreme Antarctic environments present severely limited terrestrial productivity and, consequently, soil organic matter concentrations are very low (Burkins et al., 2001). Nutrient inputs in these ecosystems have been attributed to aerial deposition (Reynolds et al., 2001), abiotic processes driven by temperature changes (Parsons et al., 2004), and microbial activity (Cary et al., 2010). The presence of phototrophic bacterial families in the Core80 community suggests its key role in carbon and energy input to the ecosystem. Therefore, *Pseudanabaena* and *Tychonema* could be involved in the CO_2_ photoassimilation and the fixation of N_2_, as observed in high-elevated ‘barren’ soils from other latitudes (Freeman et al., 2009). The presence of Chloroflexi could also be related to the CO_2_ uptake. Cyanobacteria and Chloroflexi can utilize different portions of the radiation spectrum for photosynthesis (Ley et al., 2006) and would be photosynthetically active at different microtopographic positions. The presence of *Thiobacillus* is also relevant, since all species described are obligate autotrophs (Boden, 2017), allowing an alternative energy input to the ecosystem. However, the metabolic diversity of the Core80 community does not only include autotrophic bacteria. In fact, 16 of these 20 common taxa belonged to Proteobacteria and Actinobacteria phyla, typically described for glacier retreat areas (Brown and Jumpponen, 2014). Proteobacteria is a major player in soil microbial communities around the globe, due to its high metabolic versatility. This phylum, and especially *Burkholderia*, acquires a key role in organic matter decomposition processes in Arctic soils (Tao et al., 2020), and its prevalence is related with increased soil carbon turnover upon warming in Antarctic soils (Thomson et al., 2010; Yergeau et al., 2012). Likewise, Actinobacteria groups are able to decompose organic matter, including recalcitrant polymers (Větrovský et al., 2014). Therefore, most of the Core80 described for the 4 sampling locations would be associated to processes of degradation of recalcitrant organic matter, not accessible to other microorganisms (Hawes, 2008), with a probably key role in the subsequent colonization of these oligotrophic soils.

Our results indicate that the same functional taxa (with the taxonomical resolution used in this work) are inhabiting the ecologically comparable soils sampled in this study in the Antarctic Peninsula region, regardless of the potentially different environmental constraints and independently of their geographical proximity, or duration of the ice-free period. This is consistent with previous reports from Antarctic soils, where higher levels of similarity were observed between locations with similar physico-chemical characteristics (Chong et al., 2010). Considering that the ice-free condition was acquired at different time scales at the different locations (millennia at Byers Peninsula Plateau, and few decades at Biscoe Point), it can be assumed that these taxa are first, highly transportable (most likely by the wind) and second, highly versatile. These characteristics confer those taxa a pioneer status in Antarctic soils, relating to potential colonizers in the new deglaciated soils subjected to global change, due to a wider range of stress tolerance strategies than other microorganisms (Sigler et al., 2002; Sigler and Zeyer, 2004). However, its presence in soils of such different ages, suggests that primary succession processes, in extreme ecosystems, could have an indeterminate duration.

Certainly, every sampling location, besides the central core community showed a unique fingerprint in terms of the microbial community inhabiting those soils. For instance, Biscoe showed a high diversity and abundance of Cyanobacteria which was obviously absent from other locations such as Plateau, while *Chthoniobacteraceae* were conspicuously abundant in Plateau (6.95% of the total sequences in that core community) and absent or very scarce in the other locations. We suggest that those particularities are due to local environmental characteristics as higher humidity, or different mineral composition, or even to stochastic processes for the distribution that are out of the scope of this work.

## Conclusion

In conclusion, our work is a contribution to understanding the distribution and dispersal characteristics of the microbial communities inhabiting Antarctic soils. While the heterogeneity of microbial communities can reach high levels in the soil profiles in older soils, this heterogeneity is clearer at geographical scales. However, only 20 common taxonomic groups formed the highest proportion of the ASVs sequenced from the Core80 communities, and most likely conform the Antarctic bare soil bacterial community identity. The potential metabolic diversity of the Core80 community could be linked to all the fundamental metabolic activities required for the acquisition and recycling of organic C, which would justify its presence in all the sampling locations studied.

## Data Availability Statement

The datasets presented in this study can be found in online repositories. The names of the repository/repositories and accession number(s) can be found below: BioProject ID PRJNA678471.

## Author Contributions

PA wrote the initial manuscript. All authors designed the experiments, collected the samples, analyzed the experimental data, and contributed to elaborate the final manuscript.

## Conflict of Interest

The authors declare that the research was conducted in the absence of any commercial or financial relationships that could be construed as a potential conflict of interest.

## Supplementary Material

Supplementary Plate 1 Wide angle and detail picture of every soil sampled at Antarctic Peninsula region. **(A)** Byers Peninsula Plateau. **(B)** Nunatak. **(C)** Elephant Point (Livingston Is.). **(D)** Biscoe Point.

Supplementary Table 1 Soil physical and chemical characteristics from the 4 sampling locations. The values represent the mean of all sample points analyzed.

Supplementary Table 2 Coordinates of the 4 sampling locations along the Antarctic Peninsula. The Area refers to the clusters of the samples considering the glacier front as a reference point, with (I) being the closest to it.
