# Supplementary figures and images for: Heterogeneity of Microbial Communities in Soils From the Antarctic Peninsula Region
Source: Front Microbiol. 2021 Feb 16;12:628792. doi: 10.3389/fmicb.2021.628792 (PMC7920962; doi:10.3389/fmicb.2021.628792)

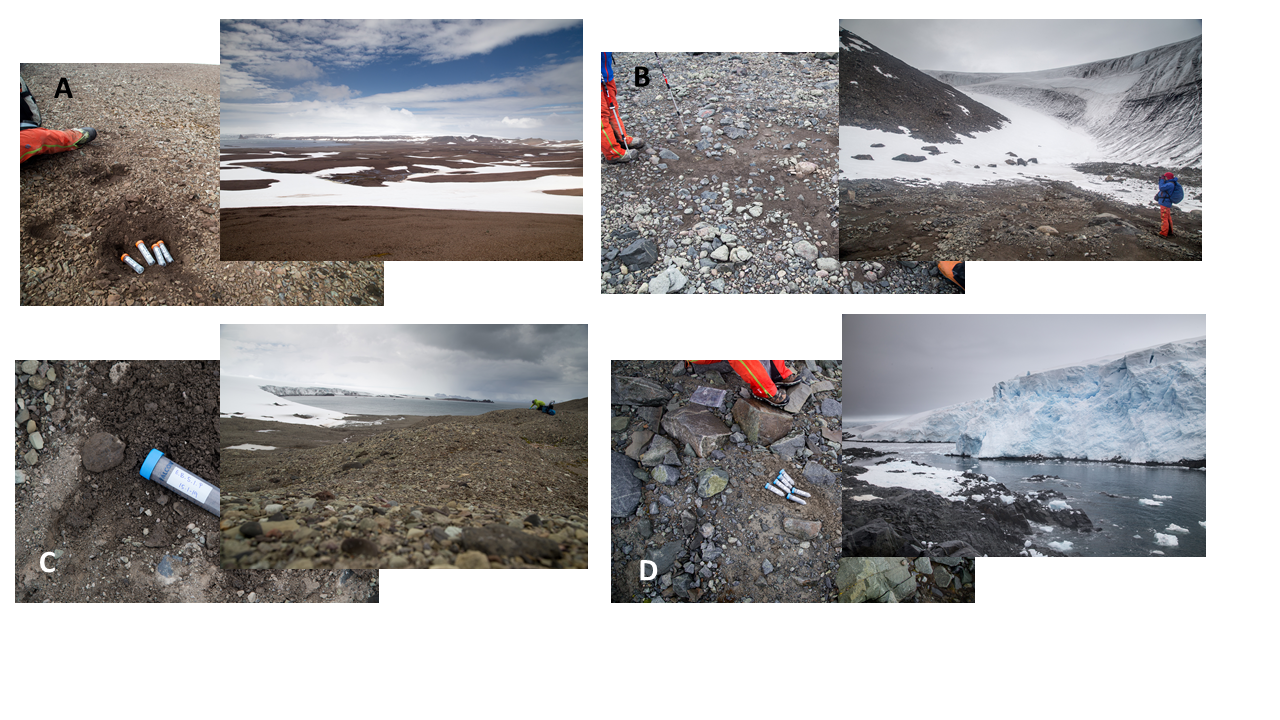

Supplement: Supplementary Plate 1 — Wide angle and detail picture of every soil sampled at Antarctic Peninsula region. (A) Byers Peninsula Plateau. (B) Nunatak. (C) Elephant Point (Livingston Is.). (D) Biscoe Point. [file Image_1.TIF]
