# Supplementary material for: Heterogeneity of Microbial Communities in Soils From the Antarctic Peninsula Region
Source: Front Microbiol. 2021 Feb 16;12:628792. doi: 10.3389/fmicb.2021.628792 (PMC7920962; doi:10.3389/fmicb.2021.628792)
Supplement: Supplementary Table 1 — Soil physical and chemical characteristics from the 4 sampling locations. The values represent the mean of all sample points analyzed. [file Table_1.docx]

**Table S1.** Soil physical and chemical characteristics from the 4 sampling locations. The values represent the mean of all sample points analysed.

| **Location** | **pH** | **C/N** | **TOM** (%p/p) | **Soil density** (g/cm^3^) | **Soil texture** |
| --- | --- | --- | --- | --- | --- |
| Plateau | 4.6 ± 0.23 | 3.9 ± 0.96 | 0.3 ± 0.11 | 1.6 ± 0.02 | Loamy sand |
| Nunatak | 6.0 ± 0.43 | 3.8 ± 1.18 | 0.2 ± 0.05 | 1.6 ± 0.04 | Sandy loam |
| Elephant | 6.8 ± 0.17 | 5.13 ± 0.35 | 0.3 ± 0.03 | 1.6 ± 0.01 | Sandy loam |
| Biscoe | 7.9 ± 0.28 | 3.8 ± 0.80 | 0.2 ± 0.04 | 1.6 ± 0.04 | Loamy sand |
