# Supplementary material for: Heterogeneity of Microbial Communities in Soils From the Antarctic Peninsula Region
Source: Front Microbiol. 2021 Feb 16;12:628792. doi: 10.3389/fmicb.2021.628792 (PMC7920962; doi:10.3389/fmicb.2021.628792)
Supplement: Supplementary Table 2 — Coordinates of the 4 sampling locations along the Antarctic Peninsula. The Area refers to the clusters of the samples considering the glacier front as a reference point, with (I) being the closest to it. [file Table_2.docx]

**Table S2.** Coordinates of the 4 sampling locations along the Antarctic Peninsula. The Area refers to the clusters of the samples considering the glacier front as a reference point, with (I) being the closest to it.

| **Plateau** | | | |
| --- | --- | --- | --- |
| Sample ID | Area | Latitute | Longitude |
| P1 | P-I | -62.6464 | -60.9661 |
| P2 | P-I | -62.6463 | -60.9662 |
| P3 | P-II | -62.6464 | -60.9676 |
| P4 | P-II | -62.6463 | -60.9677 |
| P5 | P-III | -62.6465 | -60.9691 |
| P6 | P-III | -62.6464 | -60.9691 |
| P7 | P-IV | -62.6466 | -60.9712 |
| P8 | P-IV | -62.6465 | -60.9712 |
| P9 | P-V | -62.6457 | -60.9785 |
| P10 | P-V | -62.6453 | -60.9785 |
| **Nunatak** | | | |
| N1 | N-I | -62.6695 | -60.9105 |
| N2 | N-I | -62.6694 | -60.9107 |
| N3 | N-II | -62.6697 | -60.9111 |
| N4 | N-II | -62.6696 | -60.9113 |
| N5 | N-III | -62.6701 | -60.9127 |
| N6 | N-III | -62.6701 | -60.9128 |
| **Elephant** | | | |
| E1 | E-I | -62.6808 | -60.8589 |
| E2 | E-I | -62.6817 | -60.8612 |
| E3 | E-II | -62.6811 | -60.8594 |
| E4 | E-II | -62.6818 | -60.8609 |
| E5 | E-III | -62.6811 | -60.8597 |
| E6 | E-III | -62.6822 | -60.8604 |
| E7 | E-IV | -62.6819 | -60.8597 |
| E8 | E-IV | -62.6819 | -60.8605 |
| E9 | E-V | -62.6831 | -60.8575 |
| E10 | E-V | -62.6821 | -60.8600 |
| **Biscoe** | | | |
| B1 | B-I | -64.8100 | -63.7584 |
| B2 | B-I | -64.8096 | -63.7595 |
| B3 | B-II | -64.8101 | -63.7586 |
| B4 | B-II | -64.8096 | -63.7598 |
| B5 | B-III | -64.8106 | -63.7611 |
| B6 | B-III | -64.8096 | -63.7603 |
| B7 | B-IV | -64.8101 | -63.7626 |
| B8 | B-IV | -64.8097 | -63.7608 |
| B9 | B-V | -64.8101 | -63.7667 |
| B10 | B-V | -64.8100 | -63.7631 |
